# CPEB1 modulates differentiation of glioma stem cells via downregulation of HES1 and SIRT1 expression

**DOI:** 10.18632/oncotarget.2250

**Published:** 2014-07-23

**Authors:** Jinlong Yin, Gunwoo Park, Jeong Eun Lee, Ju Young Park, Tae-Hoon Kim, Youn-Jae Kim, Seung-Hoon Lee, Heon Yoo, Jong Heon Kim, Jong Bae Park

**Affiliations:** ^1^ Specific Organs Cancer Branch, Research Institute, National Cancer Center, Goyang, Gyeonggi, Korea; ^2^ Cancer Cell and Molecular Biology Branch, Research Institute, National Cancer Center, Goyang, Gyeonggi, Korea; ^3^ Department of System Cancer Science, Graduate School of Cancer Science and Policy, National Cancer Center, Goyang, Gyeonggi, Korea

**Keywords:** Glioma stem cell, self-renewal, differentiation, CPEB1

## Abstract

Glioma stemness has been recognized as the most important reason for glioma relapse and drug resistance. Differentiation of glioma stem cells (GSCs) has been implicated as a novel approach to target recurrent glioma. However, the detailed molecular mechanism involved in the differentiation of GSCs has not yet been elucidated. This study identified CPEB1 as the key modulator that induces the differentiation of GSCs at the post-transcriptional level. Gain and loss of function experiments showed that CPEB1 expression reduced sphere formation ability and the expression of stemness markers such as Nestin and Notch. To elucidate the detailed molecular mechanism underlying the action of CPEB1, we investigated the interacting ribonome of the CPEB1 complex using a Ribonomics approach. CPEB1 specifically suppressed the translation of HES1 and SIRT1 by interacting with a cytoplasmic polyadenylation element. The expression profile of CPEB1 negatively correlated with overall survival in glioma patients. Overexpression of CPEB1 decreased the number of GSCs in an orthotopically implanted glioma animal model. These results suggest that CPEB1-mediated translational control is essential for the differentiation of GSCs and provides novel therapeutic concepts for differentiation therapy.

## INTRODUCTION

The degree of glioma malignancy is graded using a World Health Organization (WHO) consensus-derived scale of I to IV, as determined by histological features and genetic alterations [1]. Glioblastoma multiforme (GBM), the most common and biologically aggressive form of WHO grade IV gliomas, has increased in incidence and has a very poor prognosis, with an average survival of only 14~16 months despite maximal therapy [1, 2]. Treatment failure may be due to the inability of currently available therapies to eliminate cancer stem cells (CSCs), which are regarded as responsible for tumor initiation, progression, invasion, recurrence, and drug resistance [3]. CSCs are a subpopulation of cells in the tumor that have self-renewal capacity and can give rise to the various cells that comprise these tumors [4]. Glioma stem cells (GSCs) were the first cancer stem cell isolated from solid tumors [5]. Whereas one million non-GSCs were needed to generate parental tumors when implanted into immunodeficient mice, as few as 100 GSCs were required [5]. The existence of GSCs provided a plausible explanation for glioma recurrence following treatment. CD133^+^ GSCs were found to be more resistant to radiation *in vitro* than CD133^−^ cells because of induction of DNA repair pathways [6]. In addition, GSCs were found to overexpress ATP-binding cassette transporters (ABCTs) such as ATP-binding cassette sub-family G member 2 (ABCG2) to export the chemotherapeutic agent extracellularly [7].

Tumors may be treated by inducing the differentiation of CSCs. Transient *in vitro* exposure of GSCs to BMP4, which induces astroglial differentiation, abolishes their tumor initiating and infiltrating potential [8]. Thus, treatments can be designed to induce the differentiation of CSCs into more differentiated cancer cells, which lose the ability to self-renew and can respond to current therapy [9]. To date, only two anticancer drug categories have been found to affect cancer cell differentiation: retinoic acid and drugs that target tumor epigenetic factors [9]. Although induction of differentiation with *all-trans* retinoic acid has been successful in the treatment of acute promyelocytic leukemia, it has limited benefit in the treatment of solid tumors, suggesting that differentiation therapy in solid tumors may involve more complicated molecular mechanisms than promyelocytic leukemia [10]. These findings further support the importance of identifying molecular mechanisms of CSC differentiation in gliomas.

CPEB1 is a highly conserved RNA-binding protein that specifically binds to a conserved RNA sequence called the cytoplasmic polyadenylation element (CPE). The CPE is usually found in the 3′ untranslated region (3′UTR) of several key mRNAs in vertebrate germ cells, embryos, and neurons [11-13]. CPEB1, along with other cellular factors, is indirectly responsible for both translational repression and activation through regulation of polyadenylation. The CPEB1 homolog *Xenopus laevis* CPEB accomplishes these tasks through its association with several key partners, including CPSFs [14, 15], Maskin [16], Symplekin [17], Gld2 [13, 17], PARN [18], and ePAB [19]. Frog and mouse proteins induce the cytoplasmic polyadenylation of dormant mRNAs with short poly(A) tails, resulting in their translation during early developmental stages [12, 20]. Recently, the level of CPEB1 was increased during neural differentiation, with CPEB1 having a developmental role as an inducer of differentiation [21, 22]. Furthermore, CPEB1 has been implicated as a tumor suppressor in many solid tumors [23, 24].

Based on these observations, we hypothesized that regulation of CPEB1 expression may be critical for the differentiation of GSCs. Using loss and gain of function experiments, we assessed the functional significance of CPEB1 in GSCs by analyzing the CPEB1 target ribonome to elucidate the molecular details of CPEB1-mediated regulation of GSC stemness.

## RESULTS

### CPEB1 expression is inversely correlated with glioma stemness and overall survival of glioma patients

**Figure 1:**
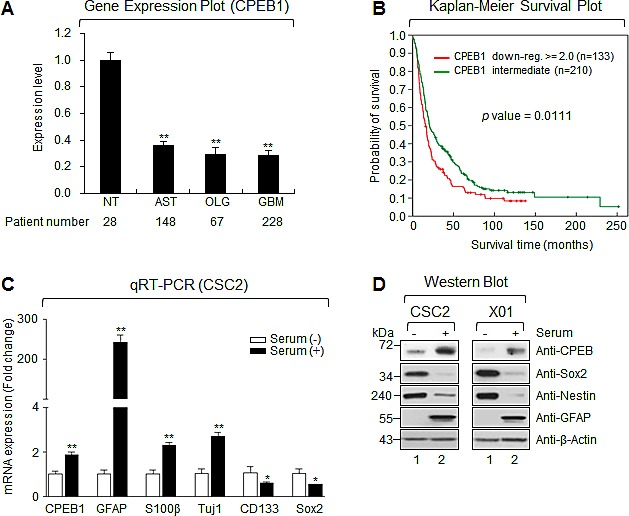
CPEB1 expression is inversely correlated with glioma stemness and overall survival of glioma patients (A) The expression level of mRNA obtained from NT (non-tumor, n = 28), AST (astrocytoma, n = 148), OLG (oligodendrocytoma, n = 67) and GBM (glioblastoma multiforme, n = 228). Data obtained from the REMBRANDT database of the National Cancer Institute. (B) Overall survival between CPEB1 down-regulated (red curve) and intermediate (green curve) patients was analyzed. Data obtained from the REMBRANDT database of the National Cancer Institute (CPEB1 down-regulated >=2-fold, n = 133; CPEB1 intermediate, n = 210; *p* = 0.0111). (C) Real-time quantitative RT-PCR (qRT-PCR) results of CPEB1, differentiation markers (GFAP, S100β, and Tuj1), stemness markers (CD133 and SOX2) were obtained from serum treated or non-treated CSC2 glioma stem cells (GSCs). Graphs are representative of three independent experiments. All error bars represent mean ± s.e.m. (n = 3). *p<0.05; **p<0.01. (D) Western blots (WB) of CPEB1, GFAP, Sox2 and Nestin in serum treated or non-treated CSC2 (left) and X01 (right) GSCs.

**Figure 2:**
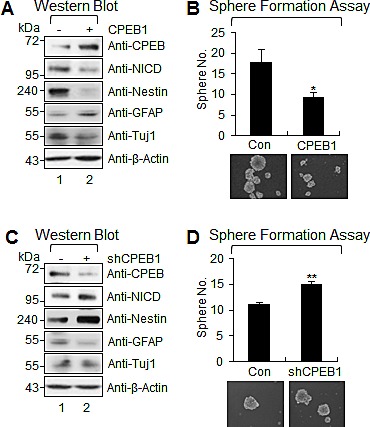
CPEB1 suppresses stemness and self-renewal ability of GSCs **(A)** WB of CPEB1, NICD, Nestin, GFAP, and Tuj1 in CSC2 infected with CPEB1-expressing lentiviral or control construct. **(B)** Sphere formation assay and its average proportion of sphere numbers in CSC2 infected with CPEB1 expressing lentivrial or control construct. Images are representative of three independent experiments. All error bars represent mean ± s.e.m. (n = 3). *p<0.05. **(C)** WB of CPEB1, NICD, Nestin, GFAP, and Tuj1 in CSC2 infected with shCPEB1-expressing lentiviral or control construct. **(D)** Sphere formation assay and its average proportion of sphere numbers in CSC2 infected with shCPEB1-expressing lentiviral or control construct. Images are representative of three independent experiments. All error bars represent mean ± s.e.m. (n = 3). **p<0.01.

To examine the possible role of CPEB1 in glioma malignancy, we analyzed the expression profile from the REMBRANDT (REpository for Molecular BRAin Neoplasia DaTa) database. CPEB1 expression was significantly lower in tumor samples from 148 patients with astrocytoma, 67 with oligodendroglioma, and 228 with GBM, than in 28 non-tumor brain tissue samples (Figure 1A). Overall survival was significantly longer in glioma patients with intermediate than low levels of CPEB1 expression (*p* = 0.0111; Figure 2B).

Due to the downregulation of CPEB1 expression in human gliomas, we assayed the levels of CPEB1 expression in patient-derived GSCs cultured in serum-free stemness and serum-containing differentiation media. GSCs lost stemness when cultured in the presence of serum [25]. Cells cultured in serum-containing media showed higher CPEB1 mRNA and protein expression, along with high levels of expression of differentiation markers (GFAP, S100β, and Tuj1) and low levels of stemness markers (Nestin, Sox2, CD133) (Figures 1C and 1D). These results indicated that CPEB1 may function as a differentiation inducer in human GSCs.

### CPEB1 suppresses stemness and self-renewal ability of GSCs

Because CPEB1 expression was increased when GSCs were cultured under differentiation conditions, we examined the functional role of CPEB1 in the stemness and self-renewal of GSCs. Since sphere formation assay and neural stemness markers such as Nestin and Notch are widely used to assess the self-renewal capacity and stemness of GSCs, we examined sphere formation ability and the expression of Nestin and Notch intracellular domain (NICD) in CPEB1-modulated GSCs [26, 27]. CPEB1 overexpression resulted in a dramatic reduction in expression of Nestin and NICD, as well as a reduction of the efficiency of sphere formation (Figures 2A and 2B). In contrast, CPEB1 depletion from CSC2 cells significantly increased Nestin expression, Notch1 cleavage, and the efficiency of sphere formation (Figures 2C and 2D). Importantly, GFAP expression positively correlated with CPEB1 but not Tuj1, suggested that CPEB1 induced differentiation of GSCs into astrocyte (Figures 2A and 2C). These results indicate that CPEB1 may suppress GSC self-renewal by inducing differentiation.

### Identification of CPEB1-associated transcripts by Ribonomics approach

As shown in Figure 2, CPEB1 suppressed self-renewal of GSCs. CPEB1 acts as a sequence specific RNA-binding protein. Moreover, CPEB1 acts to regulate polyadenylation and translation of target mRNAs, a mechanism critical for gene expression regulation. Systematic identification of CPEB1 affected target transcripts potentially involved in GSC self-renewal is therefore critical to understanding of exact regulation mechanism.

To identify target transcripts potentially modulated by CPEB1 in cells, we purified CPEB1-containing messenger ribonucleoprotein particle (mRNP) complexes from 293T cells, which stably express CPEB1 with an N-terminal tandem affinity purification tag, consisting of an S-tag (S), double FLAG epitopes (F), and a streptavidin-binding peptide (S) [28]. To test the activity of this SFS-tagged CPEB1 (SFS-CPEB1) in the formation of mRNPs and its association with target transcripts, we attempted to immunoprecipitate SFS-CPEB1 with several proteins known to interact with CPEB, such as Symplekin [17], CPSFs [14, 15], and Aurora A (AURKA) [15].

**Figure 3:**
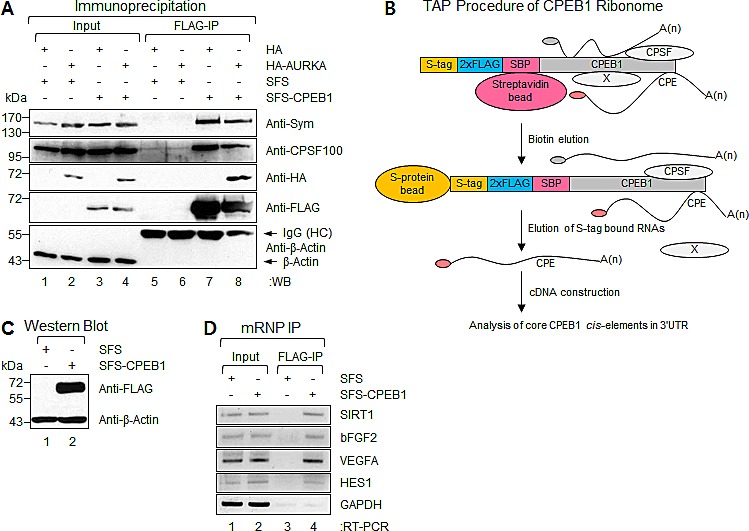
Identification of CPEB1-associated transcripts by Ribonomics approach **(A)** Plasmids encoding SFS-CPEB1 and HA-AURKA were ectopically expressed in 293T cells. SFS-CPEB1 was precipitated with anti-FLAG M2 affinity gel and the WB was performed with Symplekin, CPSF100, HA-tag, FLAG-tag, and β-Actin (negative binding control) specific antibodies, respectively. **(B)** Schematic diagram of tandem affinity purification (TAP) procedure. An S-tag, double FLAG tag, and streptavidin-binding peptide were fused at the N-terminus of CPEB1 (SFS-CPEB1). After sequential streptavidin and S-protein bead binding, the SFS-CPEB1-associated RNAs were eluted from S-protein beads. The eluted RNAs were used for cDNA library construction. X represents nonspecifically interacting protein. **(C)** Stable cell lines expressing SFS and SFS-CPEB1 were harvested and lysed, and the levels of individual samples were detected by WB. β-Actin was used as the loading control. **(D)** Physical interaction between SFS-CPEB1 and identified ribonome. Plasmids encoding SFS and SFS-CPEB1 were ectopically expressed in 293T cells. RNA-protein complexes were precipitated with anti-FLAG M2 affinity agarose gels. After IP of RNA-protein complexes, RNAs were isolated and used in semi-quantitative RT-PCR reactions with specific oligomers for SIRT1, bFGF2, VEGFA, HES1, and GAPDH (negative binding control). The PCR products were resolved on 1% agarose gel.

293T cells were cotransfected with plasmids encoding SFS-CPEB1 and HA-tagged-Aurora A (HA-AURKA) (Figure 3A). Expressed SFS-CPEB1 protein was specifically pulled down from cell lysates with anti-FLAG-M2 affinity gels. Subsequently, Western blot (WB) was performed with antibodies against the Symplekin, CPSF100, and HA-tag to detect specific protein-protein interactions. We found that SFS-CPEB1 formed a correct protein complex, indicating that SFS-CPEB1 is functionally active in cells (Figure 3A).

Using a Ribonomics strategy [29], we next attempted to identify a subset of transcripts that are potential targets of CPEB1 at the post-transcriptional level. WB of a stable clone expressing SFS-CPEB1 yielded a positive outcome at the expected molecular weight, whereas control cells were negative for this protein (Figure 3C). Using a SFS-CPEB1 expressing stable CPEB1 cell line, associated mRNPs were tandem affinity purified by sequential SBP and S-protein affinity chromatography (Figures 3B and 3C)[28].

Precipitates of the SFS-CPEB1 expressing stable CPEB1 cell line contained a detectable amount of total RNA, whereas the control cell line did not (Supplementary Figure S1A). The RNAs were used to construct a CPEB-associated transcript cDNA library, which was analyzed for the presence of essential *cis*-acting elements [CPE, Pumilio binding site (Pum), and hexanucleotide (HEX)] for cytoplasmic polyadenylation-translation control within the 3′UTR region of each clone [30]. Independently, we also analyzed several genes [vascular endothelial growth factor A (VEGFA), platelet-derived growth factor receptor beta polypeptide (PDGFRB), sirtuin1 (SIRT1), catenin beta-1 (CTNNB1), and basic fibroblast growth factor 2 (bFGF2)] essential for the proliferation and differentiation of GSCs [31-36] through the CPE prediction algorithm [30]. All of these genes were highly conserved and contained strong *cis*-acting elements in their 3′UTR regions (Supplementary Figure S2).

By using a Ribonomics strategy, from the 450 randomly picked clones, 108 had successful reads, representing 46 types of CPEB1-associated transcripts (Table 1). These transcripts included nine ribosomal protein genes (20%), 32 single represented genes (70%), and five unclassified entries (10%). When the molecular functions of these genes were classified using the DAVID Bioinformatics Resource and a SOURCE search, we found that most of the CPEB1-associated transcripts encode genes involved in various cellular pathways, including ubiquitination-related genes, genes encoding structural constituents of the ribosome, metabolic enzymes, translation initiation factors, and cellular chaperones, as well as encoding proteins involved in transcriptional regulation, including hairy and enhancer of split 1 (HES1) and YY1-associated factor 2 (YAF2) (Table 1).

**Table 1:** Ribonomic identification of CPEB1-associated transcripts

*Gene description*	*Gene symbol*	*Molecular function*	*RefSeq ID*
*Homo sapiens* ADP-ribosylation factor-like 17B	*ARL17B*	GTP-binding protein; ADP-ribosyltransferase	NM_001103154
*Homo sapiens* biogenesis of lysosomal organelles complex-1, subunit 1	*BLOC1S1*	Histone acetyltransferase activity	NM_001487
*Homo sapiens* BCL2-adenovirus E1B 19kDa interacting protein 3	*BNIP3*	Apoptosis-inducing protein	NM_004052
*Homo sapiens* bromodomain containing 7	*BRD7*	Coactivator and corepressor in chromatin modeling	NM_013263
*Homo sapiens* bromodomain containing 7 pseudogene 2	*BRD7P2*	Pseudogene; lncRNA class	NG_009641
*Homo sapiens* misc_RNA (BRD7P2), miscRNA	*BRD7P2*	Unknown	XR_015764
*Homo sapiens* chromosome 6 open reading frame 48	*C6orf48*	Major histocompatibility class? complex antigen	NM_001040438
*Homo sapiens* chromosome 14 open reading frame 2	*C14orf2*	Unknown	NM_004894
*Homo sapiens* chromosome 18 open reading frame 32	*C18orf32*	Unknown	NM_001035005
*Homo sapiens* chemokine (C-X-C motif) ligand 16	*CXCL16*	Melanoma growth stimulating activity	NM_022059
*Homo sapiens* DnaJ (Hsp40) homolog, subfamily A, member 2	*DNAJA2*	Factor in the protection	NM_005880
*Homo sapiens* DnaJ (Hsp40) homolog, subfamily C, member 1	*DNAJC1*	ATPase stimulator	NM_022365
*Homo sapiens* eukaryotic translation initiation factor 2, subunit 2	*EIF2S2*	Binding to initiator tRNA, binding to 40S ribosomal subunit	NM_003908
*Homo sapi*ens eukaryotic translation initiation factor 3, subunit M	*EIF3M*	Translation initiation	NM_006360
*Homo sapiens* eukaryotic translation initiation factor 5B	*EIF5B*	Ribosome-dependent GTPase	NM_015904
*Homo sapiens* germ cell-less homolog 1 (Drosophila)-like	*GMCL1L*	Modulating the nucleocytoplasmic trasnport	NR_003281
*Homo sapiens* golgi transport 1 homolog B (*S. cerevisiae*)	*GOLT1B*	Signal transducer	NM_016072
*Homo sapiens* hairy and enhancer of split 1, (Drosophila)	*HES1*	Transcriptional repressor	NM_005524
*Homo sapiens* interleukin 13 receptor, alpha 1	*IL13RA1*	Binding with low affinity to interleukin-13 (IL13)	NM_001560
*Homo sapiens* KIAA1797	*KIAA1797*	Unknown	NM_017794
*Homo sapiens* LIM and calponin homology domains 1	*LIMCH1*	Involved in actomyosin structure organization and biogenesis	NM_01498
*Homo sapiens* lipase A, lysosomal acid, cholesterol esterase	*LIPA*	Intracellular hydrolysis of cholesteryl esters and triglycerides	NM_000235
*Homo sapiens* similar to colon cancer-associated antigen	*LOC100132703*	Unknown	XM_001714789
*Homo sapiens* misc_RNA (LOC730004), miscRNA.	*LOC730004*	Unknown	XR_039618
*Homo sapiens* NADH dehydrogenase (ubiquinone) 1 alpha subcomplex, 2	*NDUFA2*	NADH ubiqinone oxidoreductase (Q reductase)	NM_002488
*Homo sapiens* NOL1-NOP2-Sun domain family, member 6	*NSUN6*	S-adenosyl-L-methionine-dependent methyl-transferase activity	NM_182543
*Homo sapiens* phosphodiesterase 9A	*PDE9A*	Metal ion-dependent enzymes	NM_001001580
*Homo sapiens* prolyl endopeptidase-like	*PREPL*	Serine peptidase cleaving peptide bond	NM_001042386
*Homo sapiens* ribosomal protein L39	*RPL39*	Constituent of ribosome, large subunit	NM_001000
*Homo sapiens* ribosomal protein, large, P1	*RPLP1*	Constituent of ribosome, large subunit	NM_001003
*Homo sap*iens ribosomal protein S5	*RPS5*	Component of the 40s ribosomal subunit	NM_001009
*Homo sapiens* ribosomal protein S12	*RPS12*	Component of the 40s ribosomal subunit	NM_001016
*Homo sapiens* ribosomal protein S13	*RPS13*	Component of the 40s ribosomal subunit	NM_001017
*Homo sapiens* ribosomal protein S15	*RPS15*	Component of the 40s ribosomal subunit	NM_001018
*Homo sapiens* ribosomal protein S17	*RPS17*	Component of the 40s ribosomal subunit	NM_001021
*Homo sapiens* ribosomal protein S20	*RPS20*	Component of the 40s ribosomal subunit	NM_001023
*Homo sapiens* ribosomal protein S29	*RPS29*	Component of the 40s ribosomal subunit	NM_001032
*Homo sapiens* SREBF chaperone	*SCAP*	Regulating the sterol-dependent transcription of cholesterol biosynthetic genes	NM_012235
*Homo sapiens* succinate-CoA ligase, alpha subunit	*SUCLG1*	Involved in the tricarboxylic acid cycle	NM_003849
*Homo sapiens* THAP domain containing 4	*THAP4*	Unknown; DNA binding	NM_015963
*Homo sapiens* thymosin beta 4, X-linked	*TMSB4X*	Regulator of actin polymerization	NM_021109
*Homo sapiens* translocase of outer mitochondrial membrane 20 homolog	*TOMM20*	Receptor of cytosolically synthesized mitochondrial preprotein	NM_014765
*Homo sapiens* thioredoxin-like 1	*TXNL1*	Thioredoxin-like reducing activity (protein-disulfide reduction)	NM_004786
*Homo sapiens* ubiquitin-activating enzyme E1	*UBE1*	Role in the first step of ubiquitin-proteasome pathway to activate ubiquitin	NM_153280
*Homo sapiens* ubiquitin-like 5	*UBL5*	Ubiquitin-like protein modifier	NM_001048241
*Homo sapiens* YY1 associated factor 2	*YAF2*	Binding to MYC, Inhibiting MYC-mediated transactivation	NM_005748

Specific interactions between CPEB1 and some of identified transcripts were confirmed using mRNP-immunoprecipitation (IP) experiments (Figure 3D). 293T cells were transfected with plasmids encoding SFS-CPEB1; FLAG-IP and semi-quantitative RT-PCR were then performed. As expected, VEGF, PDGFRB, SIRT1, bFGF2, and HES1, but not GAPDH, mRNAs were specifically co-precipitated with SFS-CPEB1 (Figure 3D). These results further indicated that SFS-CPEB1 formed the mRNP complex and confirmed that SFS-CPEB1 is functional in cells.

### CPEB1 modulates translation of identified target transcripts

**Figure 4:**
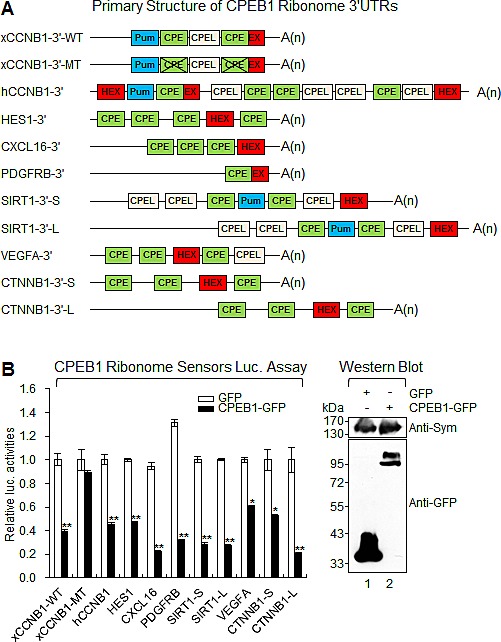
CPEB1 modulates translation of identified target transcripts **(A)** Schematic diagram of identified ribonome. CPE, cytoplasmic polyadenylation element; CPEL, cytoplasmic polyadenylation element like sequence; Pum, pumilio binding site; HEX, hexanucleotide; EX, CPE overlapped hexanucleotide. L; long variant, S; short variant. **(B)** CPEB1 repressed translation of identified ribonome. Various CPEB1 ribonome sensors were cotransfected into 293T cells along with the plasmid encoding GFP or CPEB1-GFP. The expression of CPEB1-GFP was confirmed by WB and Symplekin was used as the loading control. Data represent the mean values of at least three independent experiments performed in triplicate (*p<0.05 and **p<0.01). Error bars in the graph represent mean ± s.e.m. and the *p*-value compares the control plasmid (GFP) to CPEB1-GFP.

To assess the role of CPEB1 in regulating the identified target transcripts, we generated *Renilla* luciferase constructs (sensors), which contain the 3′UTR sequence of the target ribonome [18, 19]. All identified transcripts contained the *cis*-elements (CPE and HEX) essential for CPEB1-mediated polyadenylation and translation control (Figure 4A and Supplementary Figure S2).

To examine the activity of various sensors after CPEB1 expression, CPEB1-GFP and *Renilla* luciferase ribonome 3′UTR-encoding plasmids were cotransfected into 293T cells. As expected, expression of CPEB1-GFP but not GFP repressed the activity of all wild type sensors (Figure 4B). However, the sensor containing the CPE null *Xenopus laevis* cyclin B1 (xCCNB1) 3′UTR construct showed no response to CPEB1 expression [19]. These data strongly suggest that CPEB1 modulates translation of identified target ribonome through its association with the CPE element present in the 3′UTR sequence in cells.

### CPEB1 regulates translation of HES1 and SIRT1 mRNAs

In our data, NICD expression is regulated by CPEB1 overexpression or knockdown, and interestingly Ribonomic identified transcript HES1 is one of the target genes for Notch signaling [37, 38]. SIRT1, a NAD+-dependent histone deacetylase, is implicated in multiple biologic processes, by the modification of transcription factors. Interestingly, SIRT1 is considered as an oncogene that supports the survival of CSCs in various cancers. Therefore, we further analyzed whether HES1 and SIRT1 are potential target candidates regulated by CPEB1 at the posttranscriptional level in GSCs.

**Figure 5:**
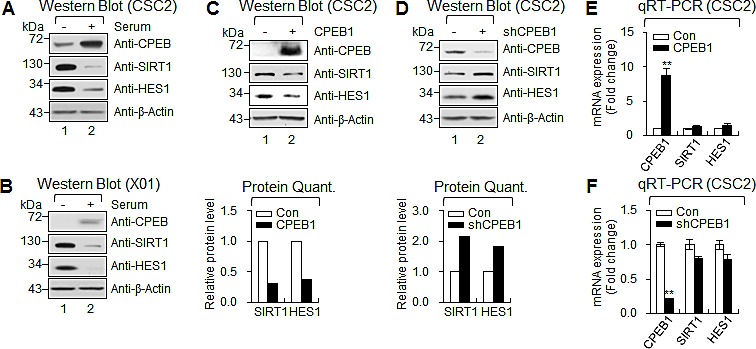
CPEB1 regulates translation of HES1 and SIRT1 mRNAs **(A and B)** WB of CPEB1, SIRT1 and HES1 in CSC2 (A) and X01 (B) with serum or without serum. **(C and D)** WB of CPEB1, SIRT1 and HES1 in CSC2 infected with CPEB1-expressing lentiviral or control construct (C) and infected with shCPEB1-expressing lentiviral or control construct (D). Expression level of SIRT1 and HES1 proteins were quantified with ImageJ software. Each protein level was normalized with that of β-Actin (loading control). **(E and F)** qRT-PCR of SIRT1 and HES1 in CSC2 infected with CPEB1-expressing lentiviral or control construct (E) and infected with shCPEB1-expressing lentiviral or control construct (F).

When cells were cultured in the presence of serum, high CPEB1 expression was inversely correlated with the levels of SIRT1 and HES1 proteins in GSCs (Figures 5A and 5B). Furthermore, CPEB1 overexpression significantly decreased SIRT1 and HES1 expression (Figure 5C). In contrast, CPEB1 depletion in CSC2 resulted in dramatic increases in both SIRT1 and HES1 expression (Figure 5D), whereas CPEB1 overexpression or depletion did not alter the expression of SIRT1 and HES1 mRNAs (Figures 5E and 5F), suggesting that the regulation occurred at the posttranscriptional level. Taken together, these findings indicate that CPEB1 functions as a translational repressor of SIRT1 and HES1 expression in GSCs.

### CPEB1 overexpression inhibits tumorigenicity of CSC2

**Figure 6:**
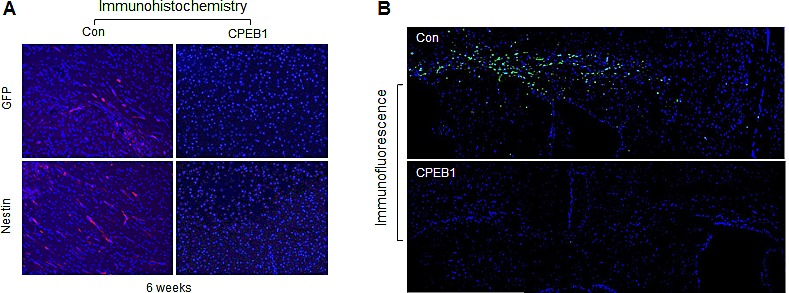
CPEB1 overexpression inhibits tumorigenicity of CSC2 model **(A)** CSC2 infected with CPEB1-GFP (right) or control (left) lentiviral construct were injected at intracranial Balb/c-nu mice. After 6 weeks, mice were sacrificed, fixed and samples were stained with Nestin and GFP. Alexa 568 to GFP and Cy3 to Nestin were used. **(B)** Free-floating assay of mouse brain tissues injected with CSC2 CPEB1 (bottom) or control (upper) cells. These cells were labeled with GFP. Representative pictures indicate mouse brain regions of corpus callusum and its surrounding structures. Nuclei were counterstained with Hoechst (blue).

Since CPEB1 induced differentiation and suppressed self-renewal ability of GSCs, we next examined its anti-tumorigenic activity in an *in vivo* mouse model. CPEB1-overexpressed GSCs, labeled with GFP, were injected into nude mouse brains. Six weeks later, the mice were sacrificed and brain samples were stained for GFP and Nestin. Tumor cells in control mice were positive for Nestin expression, but no signal was detected in mice injected with CPEB1-overexpressing GSCs (Figure 6A). GFP signals were strongly detected in the corpus callosum region of mice injected with control but not CPEB1-overexpressing GSCs (Figure 6B), strongly suggesting that CPEB1 suppressed the tumorigenicity of GSCs by inducing cell differentiation.

## DISCUSSION

The results of the present study indicate that CPEB1 functions as a potent inducer of GSC differentiation. CPEB1 overexpression decreased the self-renewal activities of GSCs, as well as downregulating stemness marker expression. Using CPEB1-associated ribonome analysis and the CPE prediction algorithm, we identified many CPEB1-related transcripts, including SIRT1 and HES1. Moreover, we found that translational control of these genes by CPEB1 regulates GSC self-renewal. The overall survival of glioma patients positively correlated with the level of CPEB1. Moreover, the concerted gain of function of CPEB1 in GSCs was found to suppress tumorigenesis and infiltrating phenotypes in an in vivo mouse model.

CPEB1 was shown to be a protein required for tumor development and may act as a tumor suppressor. The highest level of CPEB1 mRNA is observed in the reproductive system and brain, with reduced expression of CPEB1 mRNA in cancers of these tissues [23, 24]. Levels of CPEB1 are also decreased in other types of human tumors, including myeloma, gastric, breast, ovarian, and colorectal cancers [23, 24, 41, 42]. Reduced level of CPEB1 has been associated with the capacity of malignant cells to promote invasion and angiogenesis. Initially a lack of CPEB1 mediated cell immortalization, bypassing senescence of knockout mouse embryonic fibroblasts [43]. Moreover in carcinogenesis assay, papilloma formation was significantly faster in CPEB1 knockout than in wild-type animals [43]. Taken together, these results suggest that CPEB1 is a potential tumor suppressor and lack of CPEB1 potentially increases susceptibility to cancer.

Although the precise mechanism of tumor suppression by CPEB1 is still undetermined, we found that HES1 and SIRT1 were targets of CPEB1-mediated translational control. These results provide strong evidence that CPEB1 is a potent differentiation modulator of GSCs.

Recent research has highlighted the importance of CSCs in glioma malignancy [44-46]. GSCs with a potency of self-renewal and multi-lineage differentiation play an important role in glioma initiation, growth, and recurrence [47-50]. Eliminating GSCs from the bulk tumor mass may be an effective therapeutic strategy [5, 51]. Therefore, it is extremely important to understand the signaling pathways that contribute to the formation and maintenance of GSCs. Notch has been strongly implicated as a major signal in GSC maintenance. Expression of Notch regulators was found to correlate significantly with glioma grade, as well as being prognostic in glioma patients. Targeting Notch signaling was found to greatly reduce the self-renewal activity of GSCs. We found that HES1, a major downstream transcription factor for Notch signaling, was a target of CPEB1. These results indicate a novel mechanism of Notch regulation in GSCs and further support the functional significance of CPEB1 as an inducer of GSC differentiation.

SIRT1, a NAD+-dependent histone deacetylase, has been implicated in multiple biologic processes, by modifying many transcription factors, including TP53, NF-κB/p65, and FOXOs [39]. SIRT1 was recently found to be overexpressed and/or catalytically activated in tumor cells, suggesting that SIRT1 acts as a tumor promoter [40]. Moreover, Overexpression of SIRT1 has been shown to maintain CSC characteristics in different cancers. In chronic myelogenous leukemia (CML), SIRT1 overexpression was detected in CD34^+^ stem cells and implicated to increase CD34^+^ cell survival by suppressing TP53-mediated apoptosis of CML CSCs [52]. Therefore, the CPEB1-mediated down-regulation of SIRT1 may be a novel molecular mechanism by which SIRT1 is involved in GSC differentiation. CPEB1 may be functionally significant as a regulatory hub to suppress HES1 and SIRT1 translation.

CPEB1-overexpressing CSC2 showed significantly decreased proliferation and infiltration in a xenograft model, especially within the corpus callosum. CPEB1 induced differentiation of GSCs in mouse glioma model. Strikingly, these results are equivalent to glioma patient survival in REMBRANDT database. Patients with CPEB1 down-regulation have a significantly shorter overall survival than patients with intermediate levels of CPEB1 expression (*p* = 0.0111). Moreover, the level of CPEB1 mRNA is lower in gliomas (astrocytomas, oligodendrocytomas, and GBM) than in non-tumor brain tissue, further suggesting that CPEB1 plays a role as a tumor suppressor in glioma by inducing the differentiation of GSCs. These results suggest that CPEB1-mediated translational control is essential for the differentiation of GSCs and further provide novel therapeutic concepts for differentiation therapy.

## MATERIALS AND METHODS

### Cell culture

293T cells was maintained in Dulbecco's modified Eagle's medium (DMEM) supplemented with 10% fetal bovine serum (HyClone). GSCs (X01 [53], CSC2) were cultured in DMEM/F-12 supplemented with B27 (Invitrogen), epidermal growth factor (EGF, 20 ng/ml; R&D Systems) and basic fibroblast growth factor (bFGF, 20 ng/ml; R&D Systems). Differentiated GSCs were cultured in DMEM/F-12 supplemented with 10% fetal bovine serum.

### Plasmids

For the generation of pSFS-CPEB1 plasmid containing the full-length coding sequence of human CPEB1 [54], PCR was performed with cDNA from human brain cDNA library (Clontech) with the following oligomers: N-terminal fragment sense, 5′-GGGGTACCATGGCATTGTCACTGG AAGAAGAAGCAGG-3′ and antisense, 5′- GGGGATCCAGAGGCAGGAAGCTCAAGG-3′; C-terminal fragment sense, 5′-CTCTGGATCCC CTTGGGTCTGACTTGG-3′ and antisense, 5′-GCTCTAGACTAGCTGGAATCTCGGTTCTTCTGG TTCC-3′. KpnI-BamHI (N-terminal fragment) and XbaI-BamHI (C-terminal fragment) digested PCR products were inserted with pBluscript SK(-) (Stratagene). pSFS-CPEB1 was generated by ligation of NcoI-Klenow-SacII treated pBluscript SK(-)-CPEB1, and EcoRI-Klenow-SacII-CIP treated pSFS [28] together. For the generation of pEGFP-N1-CPEB1 construct, PCR was performed with pBluscript SK(-)-CPEB1 and the following oligomers; sense, 5′- GAAGATCTGCCACCATGGCATTGTCACTGGAAG-3′ and antisense, 5′-TAAACCCGGGCGCTGGAA TCTCGGTTCTTCTG-3′. The amplified DNA fragment was subcloned into pEGFP-N1 treated with BglII-XmaI-CIP. For the generation of pCMV-HA-AURKA construct, PCR was performed with cDNA from human brain cDNA library (Clontech) and the following oligomers; sense, 5′-GAAGATCTCTATGGACCGATCTAAAG AAAACTGC-3′ and antisense, 5′- GGGGTACCCTAAGACTGTTTGCTAGCTGATTC-3′. The amplified DNA fragment was subcloned into pCMV-HA (Clontech) treated with BglII-KpnI-CIP. For the generation of HRST-CPEB1-IRES-GFP construct for the lentiviral transduction, PCR was performed with pBluscript SK(-)-CPEB1 and the following oligomers; sense, 5′-TTAAAGCGGCCGCCATGGCATTGTCACT GGAAGAAGAAGC-3′ and antisense, 5′- GAAGATCTAGCTGGAATCTCGGTTCTTCTGG-3′ The amplified DNA fragment was digested with NotI-BglII and subcloned into HRST-IRES-GFP (Jong Bae Park, personal communication) treated with NotI-XhoI-CIP. For the generation of CPEB1 ribonome sensors, 3′UTR region of hCCNB1, HES1, CXCL16, PDGFRB, SIRT1-Short (SIRT1-S), SIRT1-Long (SIRT1-L), VEGFA, CTNNB1-Short (CTNNB1-S), CTNNB1-Long (CTNNB1-L) [55] which contains CPEB1 cis-elements were amplified from the human brain cDNA library (Clontech) with following oligomers: hCCNB1 sense, 5′-CTAGACTTGTAAACTTGAGTTGGAG-3′ and antisense, 5′-GTATTTGAGTATTG TTTTATTAAC-3′; HES1 sense, 5′-CTAGACTAAACA GGAACTTGAATACTGG-3′ and antisense, 5′- ATCAGTTCGAAGACATAAAAGCC-3′; CXCL16 sense, 5′-CTAGACTTACTGTGATTCCTGGCTTC-3′ and antisense, 5′-GAGAGACAAAACA AGAACTAGAG-3′; PDGFRB sense, 5′-CTAGACAACCCTGCATTGCAGGTTGG-3′ and antisense, 5′-TTGTGAGTGAGAAGCACCAGG-3′; SIRT1-S sense, 5′-ACTAGTCTACTTATAA GATGTCTCAATCTG-3′ and antisense, 5′-AAAGTCAAATGACAATTTTAATAGAC-3′; SIRT1-L sense, 5′-CTAGAGTGCAGGTACAGGAATTGTTCC-3′ and antisense, 5′-AAAGTCAAATGACAATTTTA ATAGAC-3′; VEGFA sense, 5′-CTAGAGAACCAGA TCTCTCACCAGG-3′ and antisense, 5′-GACACCAATAACATTAGCACTG-3′; CTNNB1-S sense, 5′-CTAGACAAATAGAAAATGGTCC-3′ and antisense, 5′-AATGAATTAAAAGTTTAATTC TGAACC-3′; CTNNB1-L sense, 5′- CTAGACCTGTAAATCATCCTTTAGGTAAG-3′ and antisense, 5′-AATGAATTAAAAGT TTAATTCTGAACC-3′. The amplified PCR products were subcloned into pGEM-T-easy (Promega) and digested with XbaI-NotI and then inserted into p8×Myc-Rluc [18, 56]. Construction procedure of p8×Myc-Rluc-xCCNB1-3′UTR(WT), p8×Myc-Rluc-xCCNB1-3′UTR(MT), and pLL3.7-shCPEB1 were described elsewhere. All oligomers were purchased from Cosmo Genetech (Seoul, Korea), Bioneer (Daejeon, Korea) or Macrogen (Seoul, Korea). All constructs were verified by DNA sequencing (Cosmo Genetech, Seoul, Korea).

### Sphere formation assay

Cells were plated at a density of 1,000 cells/ plate in 12 well plates and incubated in a humidified atmosphere with 5% CO_2_ at 37°C. 14 days later, plates were examined for sphere formation using an inverted microscope. The spheres with diameter >100 μm were then counted.

### Lentivirus production and transduction

293T in 100-mm plates were transfected with 6.67 μg of HRST-CPEB1-IRES-GFP or pLL3.7-shCPEB1 vector, 3.33 μg of VSV-G plasmid DNA, and 5 μg of packaging viral CMV delta 8.9 plasmid using Lipofectamine 2000 (Invitrogen). The medium was changed 6 hr after transfection. The medium containing lentivirus was harvested at 48 hr after transfection. Viral particles were concentrated and purified using a Lenti-X concentrator (Clontech). Cells were infected with lentivirus in the presence of 6 μg/ml polybrene.

### Antibodies and Western blotting

Anti-Sox2 (goat polyclonal, 1/1,000 dilution, R&D systems), anti-Nestin (mouse monoclonal, 1/1,000 dilution, BD), anti-GFAP (mouse monoclonal, 1/1,000 dilution, ImmunO), anti-NICD (rabbit polyclonal, 1/1,000, Cell Signaling), anti-HES1 (rabbit polyclonal, 1/1,000 dilution, Millipore), anti-Tuj1 (mouse monoclonal, 1/1,000 dilution, Abcam), anti-Symplekin (clone 25, mouse monoclonal, 1:1,000 dilution, BD), and anti-β-Actin (clone C4, mouse monoclonal, 1/1,000 dilution, Santa Cruz Biotech), anti-FLAG (clone M2, mouse monoclonal, 1/2,000 dilution, Sigma-Aldrich; rabbit polyclonal, 1/1,000 dilution, Cell Signaling), anti-HA (clone 3F10, rat monoclonal, 1/1,000 dilution, Roche), and anti-GFP (B-2, mouse monoclonal, 1/1,000 dilution, Santa Cruz Biotech) antibodies were used through the all WB analysis. As a secondary antibody, horseradish peroxidase-conjugated anti-rabbit (1/5,000 dilution, Vector Laboratories), anti-mouse IgG (1/5,000 dilution, Vector Laboratories), and anti-rat IgG (1/5,000 dilution, Santa Cruz Biotech) were used [57, 58].

### Ribonomics of CPEB1 and prediction of cytoplasmic polyadenylation element

Affinity purification of SFS-tagged protein mRNP complexes was performed as previously described [28]. To establish cell lines stably expressing SFS-tagged CPEB1 (SFS-CPEB1), 293T cells were transfected with plasmids encoding SFS-CPEB1 and pGK-puro. 48 hr after transfection, the cells were split at a 1:10 ratio and cultured in medium containing puromycin (Sigma-Aldrich; 2 μg/ml) for 2 weeks. The individual puromycin-resistant colonies were isolated and screened by Western blotting with anti-FLAG antibody (Sigma-Aldrich). 10 dishes (100 mm diameter) of confluent 293T cells stably expressing SFS-CPEB1 were lysed with 3.5 ml TAP lysis buffer [0.5% (v/v) Nonidet P-40, 25 mM Tris-HCl (pH >7.4), 140 mM NaCl, 10 mM NaF, 1 mM Na_3_VO_4_, 1 mM DTT, 1 mM PMSF, 10% (v/v) glycerol, 1 mM β-glycerophosphate, protease inhibitor cocktail (Roche Applied Science) without EDTA, 1mM EDTA, and RNaseOUT (Invitrogen)] on ice for 30 min. The cells were homogenized in Symplekin immunoprecipitation buffer [18] and cleared lysate was precipitated with sequential SBP and S-protein affinity gel (Novagen). Total RNA was purified from precipitated gel pellet by TRIzol reagent (Invitrogen). In all, ~1 ug of total RNA was used for making cDNA library by GeneRacer kit (Invitrogen) and amplification by 25 cycles of PCR with adapter primers. The PCR product was cloned into pBluescript II KS (-) vector (Stratagene) and transformed DH10B competent cells (Invitrogen). Nucleotide sequences from randomly chosen colonies were searched against NCBI GenBank, DAVID Bioinformatics Resources, and SOURCE search (http://source.stanford.edu). Prediction of functional CPE and the other additional controlling elements in the transcript were analyzed by use of the bioinformatics resource (http://genome.imim.es/CPE) [30].

### Semi-quantitative and real-time quantitative RT-PCR analysis

Semi-quantitative and real-time quantitative reverse transcription-polymerase chain reaction (RT- and qRT-PCR) were performed to determine mRNA levels. Total RNA was isolated from cells using TRIzol reagent (Invitrogen) according to the manufacturer's instructions. Total RNA (1~2 μg) was used as template to synthesize cDNA using M-MLV reverse transcriptase (Invitrogen) or ImProm-II reverse transcription system (Promega). qRT-PCR analysis was performed on the LightCycler 480 machine (Roche) using LightCycle 480 SYBR Green I Master Mix (Roche). The PCR primers are shown in following: CPEB1, sense 5′-GGAAGAAGAAGCAGGAAGGAT-3′ and antisense 5′-GCATCCTGCTTGTAACTGTT-3′; GFAP, sense 5′-TCTCTCGGAGTATCTGGGAACTG-3′ and antisense 5′-TTCCCTTTCCTGTCTGAGTCTCA-3′; S100β, sense 5′-TCAAAGAGCAGGAGGTTGTG-3′ and antisense 5′-TCGTGGCAGGCAGTAGTAAC-3′; Tuj1, sense 5′-ACGACGCTGAAGGTGTTCAT-3′ and antisense 5′-AGTGTGAAAACTGCGACTGC-3′; CD133, sense 5′-TTCACCTGCAGAACAGCTTC-3′ and antisense 5′-CTGTCTATTCCACAAGCAGCA-3′; Sox2, sense 5′-AACCCCAAGATGCACAACTC-3′ and antisense 5′-CGGGGCCGGTATTTATAATC-3′; SIRT1, sense 5′-TACAGTGAAGACTGTTTTCAGC-3′ and antisense 5′-TTAATAGACTTTAAAACAGTGTAC-3′; bFGF2, sense 5′-TCAAGGAAATACACCAGTTGG-3′ and antisense 5′-TGTGAAATGAGATTAGATGTGG-3′; VEGFA, 5′-TCTACATACTAAATCTCTCTCC-3′ and antisense 5′-ACGGTCCCTCTTGGAATTGG-3′; HES1 sense 5′-AACACGACACCGGATAAACC-3′ and antisense 5′-CCGCGAGCTATCTTTCTTCA-3′; GAPDH, sense 5′-GGAGTCCACTGGCGTCTTCAC-3′ and antisense 5′-GAGGCATTGCTGATGATCTTGAGG-3′. The PCR products were analyzed on the 1% agarose gel.

### Luciferase assays for various CPEB1 ribonome sensors

Various sensors were cotransfected with normalization control pGL3-Control (Promega; firefly luciferase) into 293T cells by using Lipofectamine 2000 (Invitrogen) or METAFECTENE PRO (Biontex) according to the manufacturer's instruction and previous direction [57]. After 48 hr, cells were lysed with 1 × passive lysis buffer (Promega). Aliquots of lysates were analyzed by dual luciferase reporter assay system (Promega). The sensor signal from the *Renilla* luciferase was first normalized with that from firefly (pGL3-Control; Promega). Then the signal was renormalized with that from the *Renilla* luciferase that lacked 3′UTR region (control sensor).

### Xenograft mouse model

All animal research was conducted in accordance with protocols approved by the Institutional Animal Care and Use Committee at the National Cancer Center, Republic of Korea. Cells were orthotopically transplanted following washing and re-suspension in PBS (1 × 10^5^ cells per mouse for CSC2 control and CPEB1). Cells were injected stereotactically into the left striatum of 6-week-old female Balb/c nude mice (n = 10). The injection coordinates were 2.2 mm to the left of the midline and 0.2 mm posterior to the bregma at a depth of 3.5 mm. The brain of each mouse was harvested and fixed in 4% paraformaldehyde.

### Histology and immunohistochemical staining

To allow observation of histologic features, mice were anesthetized with isoflurane and euthanized by transcardial perfusion with 10 ml of phosphate buffered saline (PBS), followed by 10 ml of 4% paraformaldehyde solution. The brains were removed, fixed with 4% paraformaldehyde for 24 hr at 4°C. For immunostaining, after the antigen retrieval process with citrate buffer (pH 6.0) and endogenous peroxidase blocking with 3% hydrogen peroxide, tissue sections were incubated in 1% BSA blocking solution (v/v) for 30 min at room temperature, then in primary antibody overnight at 4°C in a humidified chamber. For primary antibodies, we used goat antibody to GFP (Abcam, 1:500) and mouse antibody to Nestin (Abcam, 1:1,000). Sections were rinsed three times with a washing buffer (1% BSA, 0.1% cold fish skin gelatin, 0.5% Triton X-100, and 0.01 M PBS) and then incubated with secondary antibodies for 2 hr at room temperature. For secondary antibodies, we used rabbit antibody conjugated to Alexa 568 to GFP and mouse antibody conjugated to Cy3 to Nestin. To decrease non-specific Nestin signals in mouse tissue, we used the Mouse on Mouse Fluorescein kit (Vector Laboratories). Sections were mounted on slides and covered with Vectashield mounting medium with DAPI (Vector Laboratories). For free-floating immunofluorescence, after fixing brain samples in 4% paraformaldehyde solution, samples were equilibrated in a cryoprotective solution of 30% sucrose (w/v) for 24 hr. Coronal 30 μm sections were cut serially on a microtome with a freezing stage and stored in PBS at 4°C until analysis. Sections were mounted on slides and covered with Vectashield mounting medium with DAPI (Vector Laboratories).

### REMBRANDT database analysis

Expression signal values of CPEB1 gene in non-tumor brain tissues and various brain tumors and patients’ survival data grouped by CPEB1 expression levels were obtained from REMBRANDT database of the National Cancer Institute (https://caintegrator.nci.nih.gov/rembrandt/). The median values of relative probe (219578_s_at) signal of each brain tumors were normalized to that of non-tumor brain tissues.

### Statistical analysis of data

Kaplan-Meier survival plot was analyzed by Statistical Package for the Social Sciences software version 12.0 (SPSS, Chicago, IL, USA). Data are presented as the mean ± s.e.m. determined from a minimum of three independent experiments. Differences were assessed by the two-tailed Student's t-test using Excel software (Microsoft). *p<0.05 or **p<0.01 was considered statistically significant.

## SUPPLEMENTAL MATERIAL AND FIGURES


