# Supplementary material for: CPEB1 modulates differentiation of glioma stem cells via downregulation of HES1 and SIRT1 expression
Source: Oncotarget. 2014 Jul 23;5(16):6756–69. doi: 10.18632/oncotarget.2250 (PMC4196161; doi:10.18632/oncotarget.2250)
Supplement: Supplementary file 1 [file oncotarget-05-6756-s001.pdf]

CPEB1 modulates differentiation of glioma stem cells via downregulation of HES1 and SIRT1 expression

Supplementary Material

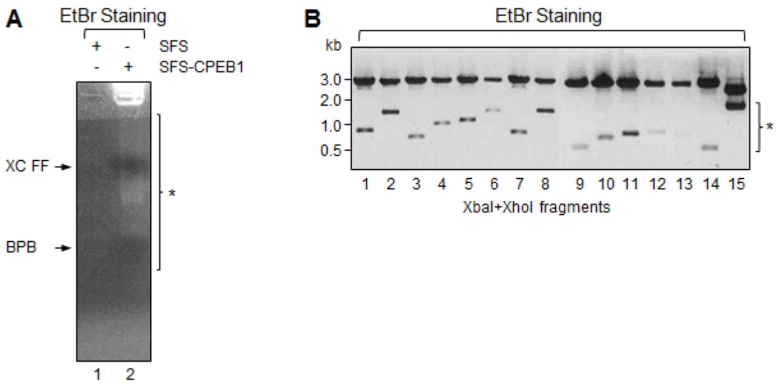

Supplementary Figure S1: (A) SFS or SFS-CPEB1 associated RNAs were purified from precipitated pellet by TRIzol reagent (Invitrogen) and then analyzed on the 1% agarose gel. XC FF, xylene cyanol FF; BPB, bromophenol blue. Asterisk represents isolated SFS-CPEB1-associated RNAs. **(B)** Some of identified independent clones were analyzed by XbaI and XhoI restriction endonuclease digestion. Asterisk represents cDNA fragments of SFS-CPEB1 ribonome.

A

Cannonical CPE: UUUUAAU, UUUUAU
Non-cannonical CPE: UUUUAAAU, UUUUACU, UUUUCAU, UUUUUGAU
Pumilio binding element: UGUAUAUA, UGUACAUA
Hexanucleotide: AAUAAA, AUUAAA

Xenopus laevis cyclin B1 (xCCNB1)
UGUUGGCACCAUGUGCUUCUGUAAAUAGUGUAUUGUGUUUUUAAUGUUUUACUGGUUUUAAUAAAGCUCAUUUUAACAUGGAAUUCAAAAAAAAAA

B

Hairy and enhancer of split 1 (HES1)
UUACUUUGUGUUUUUUUAAUUUCUAAGAAGUUACUUUUUGUAGAGAGAGCUGUAUUAAGUGACUGACCAUGCACUAUAUUUGUAUAUAUUUUAUAUGUUCAUAUUGGAUUGCGC
CUUUGUAUUAUAAAAGCUCAGAUGACAUUUCGUUUUUUACACGAGAUUUCUUUUUUAUGUGAUGCCAAAGAUGUUUGAAAAUGCUCUUAAAAUAUCUUCCUUUGGGGAAGUUUA
UUUGAGAAAAUAUAAUAAAAGAAAAAAGUAAAGGCAAAAAAAAAAAAAAAA

Chemokine (C-X-C motif) ligand 16 (CXCL16)
CAGUCCCUUGCCUACCCCAGUGAGAGUCUGAUCUUGUUUUUAUUGUUAUUGCUUUUAUUAUUAUUGCUUUUAUUAUCAUUAAAACUCUAGUUCUUGUUUUGUCUCUCCGAAAAA
AAAAAAAAAA

Thymosin, beta 4, X-linked (TMSB4X)
CCAUUUUUUUUUUUGUUCAAAUGAUUUUAAUUAUUGGAAUGCACAAUUUUUUUAAUAUGCAAAUAAAAAGUUUAAAAACUUAAAAAAAAAAAAAAA

C

Vascular endothelial growth factor A (VEGFA)
GUGACCCAGCACGGUCCCUCUUGGAAUUGGAUUCGCCAUUUUAUUUUUCUUGCUGCUAAAUCACCGAGCCCGGAAGAUUAGAGAGUUUUAUUUCUGGGAUUCCUGUAGACACAC
CCACCCACAUACAUACAUUUAUAUAUAUAUAUAUUAUAUAUAUAUAUAAAAAUAAAUAUCUCUAUUUUAUAUAUAUAAAAAAAAAAAAAAAAAAAA

Sirtuin 1 (SIRT1)
GUGUGCUAUAGAUGAUAUUUUAAAUUGAAAAGUUUGUUUUAAAUUAUUUUUACAGUGAAGACUGUUUUCAGCUCUUUUUAUAUUGUACAUAGUCUUUUAUGUAAUUUACUGGCA
UAUGUUUUGUAGACUGUUUAAUGACUGGAUAUCUUCCUUCAACUUUUGAAAUACAAAACCAGUGUUUUUUACUUGUACACUGUUUUAAAGUCUAUUAAAAUUGUCAUUUGACUU
UUUUCUGUUAAAAAAAAAAAAAAAAAAAA

Basic Fibroblast Growth Factor 2 (bFGF2)
UUCUAAUCAGAUGUAUUACUCUUAUUAUUUCUAUUGUAUGUGUUAAUGAUUUUAUGUAAAAAUGUAAUUGCUUUUCAUGAGUAGUAUGAAUAAAAUUGAUUAGUUUGUGUUUUC
UUGUCUCCCAAAAAAAAAAAAAAAAAAAA

Platelet-derived growth factor receptor, Beta polypeptide (PDGFRB)
UAUUUUUAGGACUCACGUUAACUCACAUUUAUACAGCAGAAAUGCUAUUUUGUAUGCUGUUAAGUUUUUCUAUCUGUGUACUUUUUUUUAAGGGAAAGAUUUUAAUAUUAAACC
UGGUGCUUCUCACUCACAAAAA

Catenin beta-1 (CTNNB1)
UAGACAAAUAGAAAAUGGUCCAAUUAGUUUCCUUUUUAAUAUGCUUAAAAUAAGCAGGUGGAUCUAUUUCAUGUUUUUGAUCAAAAACUAUUUGGGAUAUGUAUGGGUAGGGUA
AAUCAGUAAGAGGUGUUAUUUGGAACCUUGUUUUGGACAGUUUACCAGUUGCCUUUUAUCCCAAAGUUGUUGUAACCUGCUGUGAUACGAUGCUUCAAGAGAAAAUGCGGUUAU
AAAAAAUGGUUCAGAAUUAAACUUUUAAUUCAUUCGAUUG

Supplementary Figure S2: (A) Examples of core CPEB1 *cis*-elements. **(B)** Core CPEB1 *cis*-elements in 3'UTR of identified ribonome through Ribonomics approach. **(C)** Core CPEB1 *cis*-elements in 3'UTR of identified ribonome through bioinformatic analysis.
